# Tissue Engineering Cartilage with Deep Zone Cytoarchitecture by High‐Resolution Acoustic Cell Patterning

**DOI:** 10.1002/adhm.202200481

**Published:** 2022-07-19

**Authors:** James P. K. Armstrong, Ekaterina Pchelintseva, Sirli Treumuth, Cristiana Campanella, Christoph Meinert, Travis J. Klein, Dietmar W. Hutmacher, Bruce W. Drinkwater, Molly M. Stevens

**Affiliations:** ^1^ Department of Translational Health Sciences University of Bristol Bristol BS1 3NY UK; ^2^ Department of Materials Department of Bioengineering and Institute of Biomedical Engineering Imperial College London London SW7 2AZ UK; ^3^ Centre for Biomedical Technologies Queensland University of Technology Brisbane Queensland 4059 Australia; ^4^ Australian Research Council Training Centre in Additive Biomanufacturing Queensland University of Technology Brisbane Queensland 4059 Australia; ^5^ Australian Research Council (ARC) Training Centre for Multiscale 3D Imaging Modelling and Manufacturing (M3D Innovation) Queensland University of Technology Brisbane Queensland 4000 Australia; ^6^ ARC Training Centre for Cell and Tissue Engineering Technologies Queensland University of Technology (QUT) Brisbane Queensland 4000 Australia; ^7^ Max Planck Queensland Center for the Materials Science of Extracellular Matrices Queensland University of Technology Brisbane Queensland 4000 Australia; ^8^ Department of Mechanical Engineering University of Bristol Bristol BS8 1TR UK

**Keywords:** acoustics, cartilage, chondrocytes, patterning, ultrasound

## Abstract

The ultimate objective of tissue engineering is to fabricate artificial living constructs with a structural organization and function that faithfully resembles their native tissue counterparts. For example, the deep zone of articular cartilage possesses a distinctive anisotropic architecture with chondrocytes organized in aligned arrays ≈1–2 cells wide, features that are oriented parallel to surrounding extracellular matrix fibers and orthogonal to the underlying subchondral bone. Although there are major advances in fabricating custom tissue architectures, it remains a significant technical challenge to precisely recreate such fine cellular features in vitro. Here, it is shown that ultrasound standing waves can be used to remotely organize living chondrocytes into high‐resolution anisotropic arrays, distributed throughout the full volume of agarose hydrogels. It is demonstrated that this cytoarchitecture is maintained throughout a five‐week course of in vitro tissue engineering, producing hyaline cartilage with cellular and extracellular matrix organization analogous to the deep zone of native articular cartilage. It is anticipated that this acoustic cell patterning method will provide unprecedented opportunities to interrogate in vitro the contribution of chondrocyte organization to the development of aligned extracellular matrix fibers, and ultimately, the design of new mechanically anisotropic tissue grafts for articular cartilage regeneration.

## Graphical abstract

Engineering tissues with spatially organized cell features is essential for producing insightful biological models and effective clinical grafts. In this study, ultrasound standing waves are used to rapidly generate high‐resolution arrays of chondrocytes, which are immobilized throughout the volume of an agarose hydrogel and used to engineer hyaline cartilage with a cytoarchitecture analogous to the deep zone of articular cartilage.

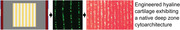

## 1 Introduction

The coordinated functions of natural tissues are enabled by the presence of conserved structural features, from the arrangement of cells to the organization of extracellular matrix components. Replicating these structural aspects is critical when seeking to engineer functional tissue grafts or relevant biological models.^[^ ^1^ ^]^ There are two overarching strategies for generating complex tissue structures. The first approach is to provide a nonuniform distribution of biochemical factors, biomaterial properties, or mechanical loads during tissue culture and maturation,^[^ ^2^, ^3^ ^]^ instructive cues that can spatially regulate processes such as cell orientation,^[^ ^4^ ^]^ cell differentiation,^[^ ^5^, ^6^, ^7^ ^]^ matrix assembly,^[^ ^6^, ^7^, ^8^ ^]^ or matrix binding.^[^ ^9^ ^]^ The second approach is to fabricate complex structures prior to tissue engineering, using methods such as self‐guided assembly,^[^ ^10^, ^11^ ^]^ directed assembly,^[^ ^11^, ^12^ ^]^ or subtractive manufacturing.^[^ ^13^ ^]^ Recent advances in these different biotechnologies have enabled the fabrication of tissues with complex macroscopic architectures^[^ ^14^, ^15^, ^16^ ^]^ or interconnected vascularized channels.^[^ ^17^, ^18^, ^19^ ^]^

An enduring challenge of this field is replicating the complex cytoarchitectures that are present in natural tissues. Recent advances in high‐resolution microfluidic/microdroplet bioprinting offer promise for engineering fine cellular features,^[^ ^20^, ^21^ ^]^ however, these mechanical processes can present shear‐induced damage during tissue assembly. These limitations have led to a growing interest in noncontact cell manipulation using optical, magnetic, or acoustic fields.^[^ ^22^ ^]^ In particular, ultrasound standing waves have been used to impart acoustic radiation forces that can remotely maneuver unlabeled populations of living cells into static acoustic pressure nodes.^[^ ^23^, ^24^ ^]^ Single pressure nodes can be used to produce dense cell aggregates for tissue engineering, an approach that has been used to form micromass cultures for cartilage tissue engineering,^[^ ^25^ ^]^ while multinodal fields can be used to form patterned cell arrays.^[^ ^26^, ^27^ ^]^ The latter approach has recently been applied to engineer complex tissues by prepatterning biomaterials with organized cell arrays. Notable examples include acoustically patterned endothelial cells,^[^ ^28^, ^29^ ^]^ adipose stem cells,^[^ ^29^ ^]^ neuroprogenitors,^[^ ^30^ ^]^ cardiomyocytes,^[^ ^31^ ^]^ and skeletal myoblasts.^[^ ^32^ ^]^

**Figure 1:**
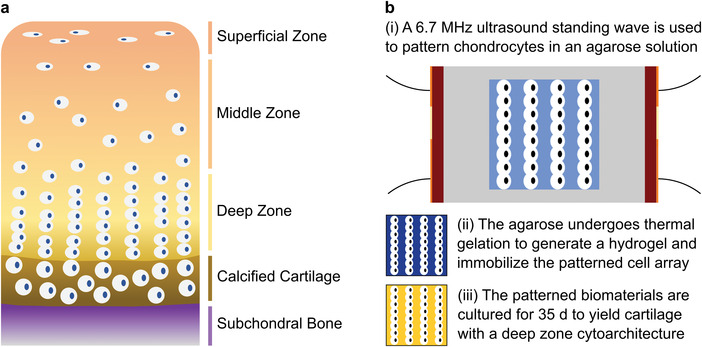
Acoustic cell patterning for engineering cartilage with deep zone cytoarchitecture. a) Schematic of the four anatomical tissue zones of articular cartilage, with the subchondral bone displayed for orientation. The deep zone accounts for ≈ 30–40% of the noncalcified cartilage tissue and contains chondrocytes that are organized into parallel aligned columns.^[^ ^33^ ^]^ b‐i) In this work, chondrocytes are suspended in a molten agarose solution (shown in light blue) and patterned using a 6.7 MHz ultrasound standing wave supplied by parallel piezotransducers (shown in red, orange indicates electrodes). ii) Thermal gelation is used to immobilize the patterned chondrocytes within an agarose hydrogel (shown in dark blue). iii) These biomaterials are then cultured into tissues with a cartilaginous extracellular matrix (shown in yellow) and a cellular microstructure resembling deep zone articular cartilage. Note that the dimensions of the cells and the cell arrays are illustrative and not to scale.

In this report, we show that acoustic cell patterning can be used to recreate the aligned cytoarchitecture of deep‐zone articular cartilage. The deep zone is one of the four classical anatomical regions of articular cartilage, present between the middle zone and the calcified zone.^[^ ^33^ ^]^ A key feature of this region is the organization of chondrocytes in uniform columns that are oriented parallel to the longitudinal axis of the joint (**Figure** **1**a). In human cartilage, these features are generally 1–2 cells wide with a spacing of around 80 µm,^[^ ^34^, ^35^ ^]^ dimensions that demand high‐resolution cell manipulation. Here, we use high‐frequency (6.7 MHz) ultrasound standing waves to acoustically pattern bovine chondrocytes into single‐cell‐width linear arrays throughout the full volume of agarose hydrogels (Figure 1b). We show that the acoustically patterned cell features are retained throughout a 35 d course of cartilage tissue engineering, with the arrays of chondrocytes producing a cartilaginous extracellular matrix with coaligned collagen fibers. We also validate that exposure to the ultrasound field has no significant effect on chondrocyte viability or metabolism, nor their capacity to engineer high‐quality hyaline cartilage. These results demonstrate the applicability of acoustic cell patterning for cartilage tissue engineering and open up new opportunities for fabricating relevant in vitro cartilage models and anisotropic tissue grafts for articular cartilage repair.

## 2 Results and Discussion

We designed and fabricated acoustic devices that were capable of patterning chondrocytes for cartilage tissue engineering. The design was broadly based on devices used in our previous work, with piezotransducers and electrical components physically separated from the cells to simplify device maintenance and sterilization.^[^ ^32^, ^36^ ^]^ Acrylic sheets were laser cut with a central square chamber (10 × 10 mm) flanked by outer cavities housing piezotransducers with wrap‐around electrodes (Figure S1, Supporting Information). The base of the device was sealed with an acetate sheet and opposing pairs of piezotransducers were connected to function generators supplying a sinusoidally varying voltage. While it was possible to drive both piezotransducer pairs in tandem, here, we operated single piezotransducer pairs independently to generate 1D bulk acoustic waves. The acoustic pressure field in the central cavity was visualized with an established model that used Huygens’ principle alongside factors such as the device geometry and ultrasound frequency.^[^ ^37^, ^38^ ^]^ Using a resonance of the piezotransducers at 6.7 MHz, this model predicted relatively uniform, 1D pressure waves with minor distortions limited to the cavity edges (Figure S2, Supporting Information).

**Figure 2:**
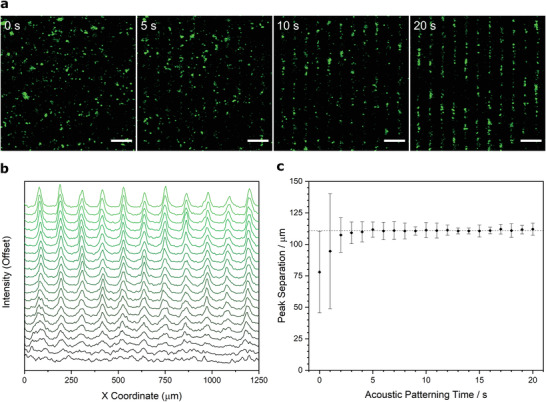
Ultrasound standing waves can pattern chondrocytes into high‐resolution arrays. a) Time‐lapse confocal fluorescence microscopy of a suspension of fixed and labeled chondrocytes (green) acoustically patterned in cell culture media using a 6.7 MHz ultrasound standing wave. The chondrocytes transition from a random distribution into an ordered linear array in under 20 s. Scale bars = 200 µm. b) Average intensity profiling performed on the time‐lapse micrographs show the gradual appearance of intensity peaks, corresponding to the formation of the organized cell array. The intensity plot is shown as an offset *y*‐axis, with timepoints from 0 s (bottom trace, black) to 20 s (top trace, green). c) The peak separation was calculated from the average intensity profiles for each timepoint (mean ± standard deviation), which shows the system rapidly converging upon the theoretical half‐wavelength spacing for a 6.7 MHz ultrasound standing wave (111 µm, indicated with dashed line).

We next sought to empirically test the acoustic patterning devices using primary articular chondrocytes, which we isolated from the femoral condyle of calf hind legs, strained into a single‐cell suspension, fixed, and fluorescently stained for visualization. We used time‐lapse confocal fluorescence microscopy to monitor the cells in a 6.7 MHz ultrasound standing wave, which showed the chondrocyte population rapidly transitioning from a randomly dispersed suspension to a parallel linear assembly (**Figure** **2**a and Video S1, Supporting Information). A fast Fourier transform performed on these micrographs confirmed the change from an isotropic cell distribution to a unidirectional periodic array (Figure S3, Supporting Information). The dynamics of this transition could also be visualized by generating temporally resolved intensity profiles, which showed periodic features appearing after just 2 s of exposure and gradually resolving into sharp peaks (Figure 2b). We identified the major intensity peaks for each of these intervals, which we then used to calculate average peak separations over time. This analysis showed the system rapidly converging on the predicted line spacing (111 µm) with an increasingly tight distribution in separation distance (Figure 2c). After 20 s of field exposure, the peak separation was 112 ± 5 µm, dimensions that are comparable to the feature spacing present in human deep‐zone cartilage (≈80 µm).^[^ ^34^, ^35^ ^]^

**Figure 3:**
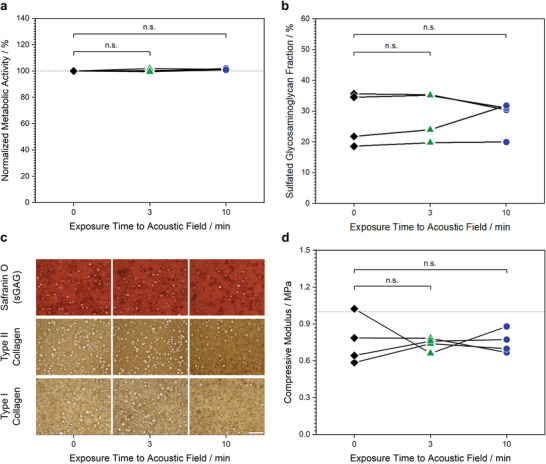
The acoustic field exposure presents no significant detrimental biological effects. Chondrocytes in cell culture media were exposed to a 6.7 MHz ultrasound standing wave for either 0, 3, or 10 min, with cell aggregates gently disrupted to study the impact of ultrasound field exposure in isolation from any acoustic patterning effects. a) Chondrocytes from each group were seeded onto tissue culture plastic and cultured for 24 h. An alamarBlue assay showed no significant difference in metabolic activity between unexposed and exposed cells (Wilcoxon signed‐rank test, *n* = 4 with connecting lines indicating that datapoints are obtained from the same biological source, n.s. = nonsignificant). b) Chondrocytes from each of the three groups were immobilized in agarose hydrogels and used to engineer cartilage for 35 d. A dimethylmethylene blue assay showed no significant difference in the level of sulfated glycosaminoglycan between the unexposed and exposed groups (Wilcoxon signed‐rank test, *n* = 4 with connecting lines indicating that datapoints are obtained from the same biological source). c) Cartilage tissues grown from each of the three groups were sectioned and either stained using safranin O to detect sulfated glycosaminoglycan, or immunostained for type II or type I collagen. No major differences in quantity or distribution were observed between the three groups. Scale bars = 200 µm. d) Cartilage tissues grown from each of the three groups were analyzed using unconfined compression testing, which showed no significant differences in compressive modulus between the unexposed and exposed groups (Wilcoxon signed‐rank test, *n* = 4 with connecting lines indicating that datapoints are obtained from the same biological source).

We next sought to assess the effect of the ultrasound standing wave on the health and function of the exposed chondrocytes. We subjected living chondrocytes suspended in culture medium to a 6.7 MHz ultrasound standing wave for either 3 or 10 min, gently mixing the cells to disrupt any patterned aggregates before comparing them with a control sample of unexposed cells (i.e., 0 min exposure). First, a small number of chondrocytes from each group were grown for 24 h on tissue culture plastic before performing an alamarBlue assay to compare cellular metabolic activity. This assay revealed no significant differences in metabolic activity between the chondrocytes exposed to ultrasound (3, 10 min) and the unexposed chondrocytes (0 min) (**Figure** **3**a). The remaining chondrocytes from each group were homogenously seeded in type VII‐A agarose hydrogels at a density of 3 × 10^7^ cells mL^–1^ and cultured for 24 h. Widefield fluorescence microscopy was then used to visualize the viable and nonviable chondrocytes after staining with calcein AM and ethidium homodimer‐1, respectively (Figure S4, Supporting Information). In each case, the vast majority of chondrocytes were viable, with no substantial differences observed between the three groups.

We next sought to assess whether ultrasound field exposure presented any long‐term implications for cartilage tissue engineering. Agarose hydrogels seeded with chondrocytes (exposed to the ultrasound standing wave field for 0, 3, or 10 min) were prepared as described above, and cultured in a medium containing transforming growth factor *β*3 (TGF *β*3), proline, ascorbic acid, dexamethasone, and insulin. These cells, materials, and growth factors have all previously been used to engineer high‐quality hyaline cartilage tissues.^[^ ^39^, ^40^ ^]^ After 35 d of tissue engineering, we lyophilized one set of tissue constructs, measured their dry mass, and then used a dimethylmethylene blue assay to evaluate the mass fraction of sulfated glycosaminoglycan. This analysis revealed high similarity between the 0 min (28 ± 9%), 3 min (29 ± 8%), and 10 min (28 ± 6%) groups (Figure 3b). Another set of tissue constructs was fixed, sectioned, and histologically stained to visualize the quantity and distribution of cartilaginous extracellular matrix produced over the 35 d culture period (Figure 3c). Safranin O revealed the three groups to have a comparable presence of sulfated glycosaminoglycan, consistent with the results from the dimethylmethylene blue assay, while immunostaining showed a similar result for type II collagen. These two biomolecules are the primary extracellular matrix components of hyaline articular cartilage. We also observed a similar quantity and distribution of type I collagen, a fibrocartilage protein that is not characteristic of native hyaline cartilage but is commonly present in tissue‐engineered cartilage.^[^ ^41^ ^]^

Sulfated glycosaminoglycans and type II collagen are the primary contributors to the mechanical properties of native articular cartilage.^[^ ^41^ ^]^ Given the observed biochemical and histological similarity, we expected that the mechanical properties would also be comparable between the three groups. We thus performed repeated cycles of unconfined compression on engineered cartilage tissues from each group. The cycles were highly consistent, and the 0–5% strain range of the stress–strain plots could be fitted via linear regression. This confirmed that there was a high similarity between the compressive Young's moduli of the 0 min group (0.76 ± 0.20 MPa), 3 min group (0.74 ± 0.05 MPa), and 10 min group (0.75 ± 0.09 MPa) (Figure 3d).

**Figure 4:**
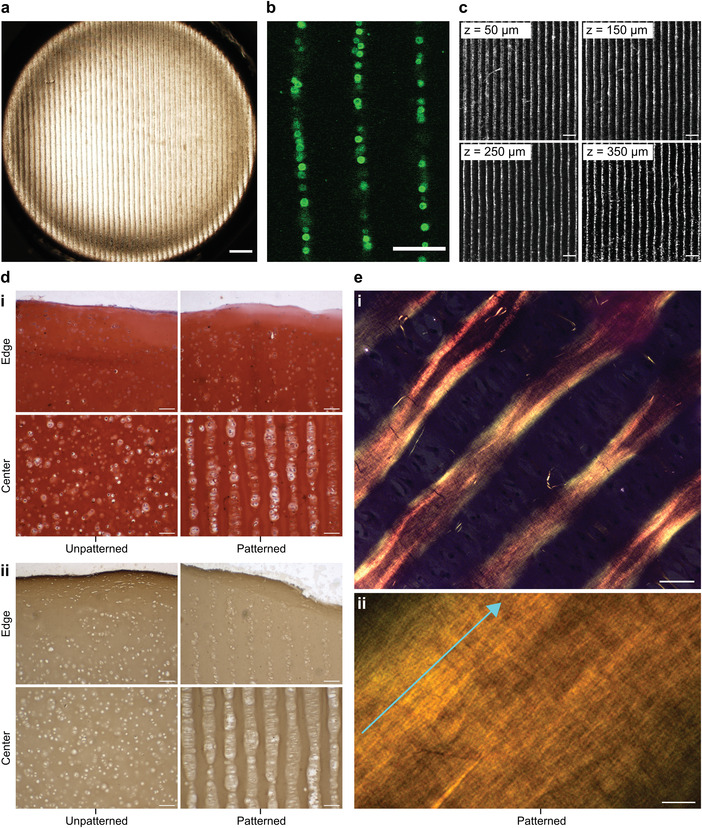
Acoustic cell patterning can be used to engineer hyaline cartilage with deep zone cytoarchitecture. a) Chondrocytes were patterned in 1.5% (w/v) agarose hydrogels using a 6.7 MHz ultrasound standing wave. Low‐magnification brightfield microscopy shows the extent and uniformity of the acoustic cell patterning across the agarose hydrogel. Scale bar = 500 µm. b) High‐magnification confocal fluorescence microscopy shows the patterned chondrocytes, labeled with a fluorescent membrane stain (green) for visualization. The patterned features were predominantly single‐cell width, which is analogous to the cellular organization of deep zone articular cartilage. Scale bar = 100 µm. c) Confocal fluorescence microscopy showing the arrays of fluorescently labeled chondrocytes (white), captured at different z‐planes of the acoustically patterned agarose hydrogel. The focal height of each *z*‐plane is indicated in the top‐left corner of each image. Scale bars = 200 µm. d) The patterned cartilage and unpatterned controls were sectioned and i) stained for sulfated glycosaminoglycan using safranin O and ii) immunostained for type II collagen. This revealed extensive matrix deposition and a cytoarchitecture reminiscent of deep zone articular cartilage throughout the majority of the tissue, with only the edges of the tissue exhibiting a disorganized distribution of chondrocytes. Scale bars = 100 µm. e) The patterned cartilage was also stained with picrosirius red and imaged using a polarized microscope at i) low magnification and ii) high magnification. The strong birefringence suggested the presence of collagen fibers, which were oriented parallel with the direction of the patterned chondrocytes (indicated by the light blue arrow). Scale bar for i) = 50 µm, scale bar for ii) = 5 µm. For a low magnification image of safranin O staining see Figure S6 (Supporting Information), for picrosirius red staining of the unpatterned control see Figure S7 (Supporting Information).

Collectively, these analyses indicated that chondrocytes subjected to ultrasound standing waves at the chosen parameters (<10 min, 6.7 MHz) have unimpaired cellular metabolism, membrane viability, and capacity for generating high stiffness, cartilaginous extracellular matrix. Therefore, we sought to investigate whether we could use these same field parameters to generate an aligned cell structure that was analogous to the cytoarchitecture of deep zone articular cartilage. Chondrocytes were mixed with molten 1.5% (w/v) type VII‐A agarose at a density of 3 × 10^7^ cells mL^–1^ and then subjected to 3 min of acoustic cell patterning (6.7 MHz). The system was allowed to cool from 37 °C to room temperature in order to immobilize the cell arrays by thermal gelation. Using this protocol, we were able to reproducibly generate agarose hydrogels (10 mm × 10 mm × 2 mm) containing uniform arrays of chondrocytes. Low‐resolution brightfield microscopy showed cells patterned across the breadth of the hydrogel (**Figure** **4**a), while high‐resolution confocal fluorescence microscopy revealed these arrays to be uniformly separated and 1–2 cells in width (Figure 4b). Importantly, the 1.5% (w/v) agarose had a sufficient polymer density to suspend the chondrocytes against gravity, thus enabling periodic cell arrays to be formed throughout the entire volume of the hydrogel (Figure 4c).

The agarose hydrogels laden with aligned chondrocytes were then put through a 35 d course of cartilage tissue engineering using the protocols described above. This culture produced tissues with an opaque appearance and a smooth exterior surface, visually similar to the unpatterned tissue controls. There were also biochemical similarities between the two groups, with a similar level of sulfated glycosaminoglycan fraction observed for the patterned tissues (47 ± 7%) and the unpatterned controls (45 ± 6%) (Figure S5, Supporting Information). This was supported by safranin O staining and immunostaining, which qualitatively suggested a similar level of sulfated glycosaminoglycans and type II collagen in sections taken from patterned and unpatterned cartilage tissues (Figure 4d). Notably, however, the cartilage engineered from acoustically patterned hydrogels retained their cell organization, exhibiting parallel lines of chondrocytes that were typically 1‐2 cells wide with a separation of ≈120 µm. The small increase in feature separation from the patterned agarose hydrogels (*D*_s_ ≈ 112 µm) was attributed to the bulk expansion that typically occurs over five weeks of extracellular matrix secretion and tissue maturation. The patterned features spanned the breadth of the engineered tissue (Figure S6, Supporting Information), with the exception of the outermost tissue regions that exhibited a disordered cytoarchitecture. No evidence of cellular organization was observed in the unpatterned controls.

We next sought to determine whether the patterned cytoarchitecture might direct extracellular matrix organization. A common histological approach to assess collagen networks in cartilage is to stain tissue sections with picrosirius red, which enhances the birefringence of collagen fibers under crosspolarized light.^[^ ^42^ ^]^ Using this method, we observed a striking fibrillar network present in the patterned cartilage, with collagen fibers aligned parallel with the lines of chondrocytes (Figure 4e). The level of observed birefringence varied throughout the tissue, however, we consistently observed the highest intensities in the regions immediately adjacent to the cell lines. The unpatterned tissues exhibited a reduced birefringence and no overall directionality to fiber alignment (Figure S7, Supporting Information). Taken together, these results conclusively indicate that acoustic cell patterning can organize living cells into high‐resolution cytoarchitecture in mature engineered tissues, and recreate some of the anisotropic cellular and fiber alignment that is present in the deep zone of human articular cartilage.^[^ ^34^, ^35^ ^]^

The anisotropic mechanical properties of native cartilage arise from the directional organization of extracellular matrix fibers. Therefore, an important future study will be to assess whether the observed collagen alignment leads to anisotropic tissue mechanics in the patterned cartilage constructs. This will necessitate a redesign of the patterning device to generate tissues with dimensions that allow mechanical loads to be applied both orthogonally and in parallel with the direction of the aligned fibers. Another important future study will be to design methods that can produce deep zone cartilage that is fully integrated with the other anatomical zones of articular cartilage (zone of calcified cartilage, middle zone, superficial zone). Orthogonal co‐patterning of superficial zone and deep zone chondrocytes would require significantly more complex acoustic pressure fields than presented in this study and would likely necessitate the development of more sophisticated patterning devices.^[^ ^43^ ^]^ However, results from this study show that it is possible to generate adjacent regions of patterned and unpatterned chondrocytes. It may therefore be possible to direct ultrasound standing waves across the mid portion of a hydrogel precursor to replicate the transitions between the patterned deep zone and the adjacent unpatterned regions (zone of calcified cartilage, middle zone).

Furthermore, the general sequence of acoustic cell patterning followed by gelation appears to be broadly compatible with component redistribution strategies that have been developed for stem‐cell‐based osteochondral tissue engineering.^[^ ^2^, ^3^ ^]^ For example, we recently showed that density‐based fluid demixing can be used to present morphogen gradients across gelatin methacryloyl (GelMA)‐based hydrogels laden with human mesenchymal stem cells (hMSCs), an approach that results in spatially‐resolved differentiation of stem cells into either osteoblasts or chondrocytes.^[^ ^7^ ^]^ We thus sought to test whether we could acoustically pattern hMSCs in a GelMA hydrogel. We mixed a suspension of hMSCs with a prepolymer solution of 5% (w/v) GelMA and acoustically patterned for 10 min at 37 °C. A 2 min application of ultraviolet light irradiation was used to photocrosslink the polymer and form a GelMA hydrogel. Cell membrane viability staining and confocal fluorescence microscopy after gelation showed hMSCs patterned in the GelMA hydrogel, a cytoarchitecture that was not observed in the controls (Figure S8, Supporting Information). This broadens the potential applicability of acoustic patterning for different cartilage tissue engineering protocols and opens up opportunities to integrate this method with existing protocols for stem‐cell‐based biofabrication of osteochondral tissue grafts.

## 3 Conclusions

This work demonstrates that a brief period of ultrasound exposure (3 min) can be used to spatially organize chondrocytes for an extensive course of tissue engineering (5 weeks). The patterned cell features were maintained throughout tissue maturation, moreover, we observed evidence of collagen fibers coaligned with the chondrocyte arrays. The long‐term pattern fidelity coupled with the absence of any observed detrimental biological effects from the ultrasound exposure makes acoustic cell patterning a valuable biotechnology for cartilage tissue engineering. The ability to reliably recreate deep zone cartilage cytoarchitecture will enable the production of in vitro models that can be used to investigate the complex interplay between chondrocyte organization and extracellular matrix assembly. In particular, understanding the contribution of chondrocyte organization to the orientation of secreted extracellular matrix fibers would open up new opportunities for engineering aligned cartilage tissue grafts with anisotropic architecture and unidirectional mechanical properties. Combining this mechanistic understanding alongside more complex acoustic devices represents a promising next step toward building full‐thickness cartilage tissue grafts that exhibit physiological organization in both cells and matrix fibers.

## 4 Experimental Section

### General

Unless stated otherwise, chemicals were purchased from Merck, experiments were run at room temperature, and deionized water was used at 18.2 MΩ cm. All live‐cell experiments were performed using sterilized components under sterile culture conditions.

### Isolation of Chondrocytes

Acoustic cell patterning and tissue engineering were performed using primary bovine articular chondrocytes obtained from the femoral condyle of hind legs from 4 w calves, with tissues sourced from a local abattoir. All animal tissue procurement protocols were performed with institutional approval from Imperial College London. The explanted cartilage was cut into fine pieces (2–3 mm) and digested for ≈16 h at 37 °C in low‐glucose Dulbecco's modified Eagle's medium (DMEM) with GlutaMAX and pyruvate (Gibco) supplemented with 1% (v/v) penicillin/streptomycin solution (Gibco) and 0.2% (w/v) type II collagenase (Gibco). The tissue digest was passed through a 100 µm nylon mesh sterile cell strainer (Thermo Fisher Scientific) to remove any undigested cartilage. The resulting chondrocyte suspension was then centrifuged at 550 × *g* for 12 min, resuspended in fresh DMEM, centrifuged at 450 × *g* for 8 min, resuspended in fresh low‐glucose DMEM, and then centrifuged at 450 × *g* for 8 min. The resulting pellet was then resuspended in fresh low‐glucose DMEM and then passed through a 40 µm nylon mesh sterile cell strainer (Thermo Fisher Scientific) to obtain a chondrocyte suspension free of cellular aggregates. The isolated chondrocytes were either: i) fixed for 20 min in a 4% (w/v) solution of formaldehyde in phosphate‐buffered saline (PBS, Gibco), washed twice with PBS, and stained for 20 min using a 4 µg mL^–1^ solution of Wheat Germ Agglutinin Alexa Fluor 488 Conjugate (Thermo Fisher Scientific) in PBS, or ii) used immediately as live cells for cartilage tissue engineering.

### Fabrication and Operation of Acoustic Devices

12‐mm thick acrylic sheets were laser cut with five cavities, as per the design provided in Figure S1 (Supporting Information). NCE51 plate piezotransducers (12 mm length, 4 mm width, 1 mm thickness) with silver wrap‐around electrodes (Electro‐Mag Ltd) were affixed to the inside bottom edge of the four outer cavities using superglue. A thin acetate sheet was affixed to the bottom of the device using Marabu Fixo Gum Rubber Cement. Insulated electrical wires were soldered to each of the electrodes and a parallel piezotransducer pair was connected to an Aim TTi TG120 20 MHz Function Generator supplying a 6.7 MHz sinusoidal voltage waveform. The frequency and peak‐to‐peak voltage (either 9 V_pp_ or 20 V_pp_) were measured using an Iso‐Tech IDS6052‐U Digital Storage Oscilloscope. To ensure effective dissipation of heat, the cavities housing the piezotransducers were filled with deionized water during device operation.

### Modeling of Acoustic Pressure Fields

The pressure field generated in the central cavity of the acoustic patterning devices was modeled using Huygens’ principle.^[^ ^38^ ^]^ Modeling was performed using a published script, which was adapted and run using MATLAB software.^[^ ^37^ ^]^ The model used the dimensions described in Figure S1 (Supporting Information) and the configuration shown in Figure S2a (Supporting Information), with two piezotransducers each positioned 9.5 mm from the center of the central cavity. The frequency was set to 6.7 MHz, while the density of the culture media (1000 kg m^–3^) and the speed of sound in culture media (1500 m s^–1^) were approximated to literature values for water.^[^ ^44^ ^]^ The chondrocyte radius (7.5 µm) was approximated through brightfield microscopy.

### Live Imaging of Acoustic Cell Patterning

A suspension of fixed and stained chondrocytes in PBS was added to the central cavity of the acoustic patterning device and imaged using a Leica SP5 inverted confocal fluorescence microscope, with images captured every 1 s. After 5 s, the cells were subjected to an ultrasound standing wave (6.7 MHz, 9 V_pp_) with images continuing to be captured every 1 s. ImageJ software (National Institutes of Health, USA) was used to export images from time‐lapse stacks and perform Fast Fourier Transform and profile plot analyses for the 20 s following ultrasound field exposure.

### Patterning Chondrocytes in Agarose Hydrogels

To provide a nonadherent coating for cell patterning, 200 µL of 3% (w/v) molten UltraPure Agarose 1000 (Thermo Fisher Scientific) was added to the central cavity of an acoustic patterning device and allowed to gel by cooling to room temperature. A suspension of fixed, stained chondrocytes was added to an equivalent volume of molten type VII‐A agarose, with both solutions mixed at 37 °C. The final cell density was 3 × 10^7^ cells mL^–1^ and the final agarose concentration was 1.5% (w/v). 200 µL of this solution was added to the central cavity of the acoustic patterning device and a single piezotransducer pair was driven at a frequency of 6.7 MHz and an amplitude of 20 V_pp_. The acoustic field was maintained for 3 min, during which time the solution was cooling to room temperature. After 1 h, the patterned agarose hydrogel was removed from the device for imaging. Low magnification brightfield images were captured with an Olympus BX51 widefield microscope, while high magnification images were obtained using a Leica SP5 inverted confocal fluorescence microscope.

### Engineering Acoustically Patterned Cartilage Tissue

All components were sterilized, with the device washed in 70% (v/v) ethanol and irradiated for 20 min with 365 nm ultraviolet light using a UVItec LF‐206.LS lamp. To provide a nonadherent coating for cell patterning, 200 µL of 3% (w/v) molten UltraPure Agarose 1000 was added to the central cavity of an acoustic patterning device and allowed to gel by cooling to room temperature. A suspension of live chondrocytes was added to an equivalent volume of molten type VII‐A agarose, with both solutions mixed at 37 °C. The final cell density was 3 × 10^7^ cells mL^–1^ and the final agarose concentration was 1.5% (w/v). 200 µL of this solution was added to the central cavity of the acoustic patterning device and a single piezotransducer pair was driven at a frequency of 6.7 MHz and an amplitude of 20 V_pp_. The acoustic field was maintained for 3 min, during which time the solution was cooling to room temperature. After 1 h, the patterned agarose hydrogel was removed from the device and transferred to a cartilage tissue engineering media, which consisted of high‐glucose DMEM with GlutaMAX and pyruvate (Gibco), 1× (v/v) penicillin/streptomycin, 1× modified Eagle's medium nonessential amino acids (Gibco), 1× insulin‐transferrin‐selenium (ITS‐G, Gibco), and 50 µg mL^–1^ l‐proline with freshly supplemented 10 ng mL^–1^ recombinant human transforming growth factor *β*3 (R&D Systems), 100 × 10^–9^ m dexamethasone, 80 × 10^–6^ m l‐ascorbic acid, and after 7 d of culture, 10 µg mL^–1^ recombinant human insulin. The media was changed three times a week for a total of 35 d, before harvesting the tissues for analysis of sulfated glycosaminoglycan (see Safranin O Staining and Dimethylmethylene Blue Assay sections below) and collagen (see Picrosirius Red Staining and Immunostaining sections below). Unpatterned tissue controls were generated using an identical protocol but without any acoustic field exposure.

### Field Exposure Studies

To provide a nonadherent coating for cell patterning, 200 µL of 3% (w/v) molten UltraPure Agarose 1000 was added to the central cavity of an acoustic patterning device and allowed to gel by cooling to room temperature. A 200 µL suspension of chondrocytes was added to the central cavity of the acoustic patterning device and a single piezotransducer pair was driven at a frequency of 6.7 MHz and an amplitude of 20 V_pp_ for either 0, 3, or 10 min. Each group of chondrocytes was maintained in the acoustic devices for the full 10 min, even when the field was turned off. The chondrocytes were gently mixed using a 1 mL micropipette and counted using a hemocytometer. Five experiments were then performed. i) *Cell Metabolism Assay*. 5 × 10^5^ chondrocytes from each group were seeded on tissue culture plastic and cultured in low‐glucose DMEM with GlutaMAX and pyruvate, supplemented with 1% (v/v) penicillin/streptomycin. After 24 h, the culture media was exchanged for low‐glucose DMEM with GlutaMAX and pyruvate, supplemented with 1% (v/v) penicillin/streptomycin and 10% (v/v) alamarBlue (Invitrogen). After 6 h of culture, aliquots were transferred to a 96 well plate and the fluorescence was measured using a microplate reader (excitation = 570 nm, cut‐off = 590 nm, emission = 610 nm). The absorbance values were compared against the linear region of a standard curve that was generated using 0, 5 × 10^4^, 1 × 10^5^, 5 × 10^5^, and 1 × 10^6^ chondrocytes that were cultured for the same conditions as above. Four paired biological repeats were used for each group, with statistical comparison performed using a Wilcoxon signed‐rank test. ii) *Cell Viability Assay*. Chondrocytes from each group were seeded at a density of 3 × 10^7^ cells mL^–1^ in 1% (w/v) type VII‐A agarose hydrogels and cultured in cartilage tissue engineering media. After 24 h, the hydrogels were manually bisected to enable the chondrocytes at the center of the hydrogel to be stained for 20 min at 37 °C using a LIVE/DEAD cell viability assay (Invitrogen), using the recommended dilutions. The samples were transferred to phenol‐free DMEM supplemented with 20 × 10^–3^ m (4‐(2‐hydroxyethyl)‐1‐piperazineethanesulfonic acid) (HEPES) buffer and imaged using an Olympus BX51 widefield microscope. iii) *Dry Mass and Sulfated Glycosaminoglycan Measurements*. Chondrocytes from each group were seeded at a density of 3 × 10^7^ cells mL^–1^ in 1% (w/v) type VII‐A agarose hydrogels and cultured in cartilage tissue engineering media. After 35 d, the constructs were harvested, digested, and used to measure sulfated glycosaminoglycan content (see Dimethylmethylene Blue Assay section below). iv) *Histology*. Chondrocytes from each group were seeded at a density of 3 × 10^7^ cells mL^–1^ in 1% (w/v) type VII‐A agarose hydrogels and cultured in cartilage tissue engineering media. After 35 d, the constructs were harvested, fixed, and stained for sulfated glycosaminoglycans, type II collagen, and type I collagen (see Safranin O Staining and Immunostaining sections below). v) *Mechanical Testing*. Chondrocytes from each group were seeded at a density of 3 × 10^7^ cells mL^–1^ in 1% (w/v) type VII‐A agarose hydrogels and cultured in cartilage tissue engineering media. After 35 d, the constructs were harvested for unconfined compression testing using a TA Instruments ElectroForce 3200 equipped with an Omega LCFD‐5 load cell. Five compression cycles of 1 N load were applied to the tissues with a 60 s dwell time in between each cycle. The resulting load‐displacement curves were used to generate stress‐strain curves based on the tissue dimensions (diameter range = 5.83–6.14 mm, height range = 1.76–1.96 mm, measured prior to testing). The compressive moduli were calculated by fitting the 0–5% strain range of compression cycles 2–5 using linear regression. Four paired biological repeats were used for each group, with statistical comparison performed using a Wilcoxon signed‐rank test.

### Dimethylmethylene Blue Assay

The cartilage tissue constructs were lyophilized and weighed to obtain values for the dry tissue mass. The cartilaginous matrix was then digested using a digestion solution (2 mg mL^–1^ TPCK‐treated trypsin, 1 × 10^–3^ m iodoacetamide, 1 × 10^–3^ m ethylenediaminetetraacetic acid, and 10 µg mL^–1^ Pepstatin A in 50 × 10^–3^ m tris(hydroxymethyl)‐aminomethane buffer at pH 7.6). One volume of digestion solution was added to the tissues, which were incubated overnight at 37 °C and 100 RPM using a ThermoMixer (Thermo Fisher Scientific). A second volume of digestion solution was then added to the tissues, which were incubated for 3 h at 65 °C and 100 RPM using a ThermoMixer, with vortex‐mixing every 10 min for the first hour. The trypsin activity was then inhibited by boiling the samples for 15 min at 100 °C using a ThermoMixer. The samples were centrifuged at 20 000 *g* for 2 min, then the supernatant was transferred into a fresh microcentrifuge tube and stored at 4 °C until analysis. To determine the sulfated glycosaminoglycan content, the samples were diluted 1 in 200 using deionized water and then transferred in duplicate to a 96 Well Clear Polystyrene Microplate (Corning). Eleven standards of chondroitin sulfate were dissolved in deionized water and plated in duplicate across a concentration range of 0–50 µg mL^–1^. A working solution (16 mg L^–1^ 1,9‐dimethylmethylene blue, 3.04 g L^–1^ glycine, 2.37 g L^–1^ sodium chloride) was added to the standards and samples at a 12.5 volume excess, then the absorbance at 525 nm was measured immediately using a microplate reader. The chondroitin sulfate standards were used to estimate the sulfated glycosaminoglycans present in the samples, which were then normalized by their dry mass. Paired biological repeats were used for each group, with statistical comparison performed using a Wilcoxon signed‐rank test.

### Safranin O Staining

The cartilage tissue constructs were washed in PBS, fixed in 4% (v/v) formaldehyde overnight, and then transferred to 70% (v/v) ethanol before paraffin embedding and sectioning (10 µm thick sections). Paraffin was removed using a 6 min immersion in Histoclear (Geneflow Ltd) before rehydrating the samples by sequential 2 min immersions in 100% (v/v) ethanol, 90% (v/v) ethanol, 70% (v/v) ethanol, and deionized water. Sections were stained for 4 h in a 0.02% (w/v) solution of Fast Green FCF, dipped three times in a 1% (v/v) aqueous solution of acetic acid, stained for 6 min in a 0.1% (w/v) solution of Safranin O, dipped 10 times in 95% (v/v) ethanol, dipped 20 times in 100% (v/v) ethanol, then immersed for 1 min in 100% (v/v) ethanol. The stained sections were then dehydrated by sequential 2 min immersions in 70% (v/v) ethanol, 90% (v/v) ethanol, 100% (v/v) ethanol, then immersed twice for 3 min in Histoclear, then mounted with coverslips and DPX Mountant (VWR).

### Picrosirius Red Staining

The cartilage tissue constructs were washed in PBS, fixed in 4% (v/v) formaldehyde overnight, and then transferred to 70% (v/v) ethanol before paraffin embedding and sectioning (10 µm thick sections). Paraffin was removed using Histoclear before rehydrating the samples by sequential 2 min immersions in 100% (v/v) ethanol, 90% (v/v) ethanol, 70% (v/v) ethanol, and deionized water. Sections were stained for 2 min in Celestin Blue solution (Solmedia), rinsed in tap water, stained for 2 min in hematoxylin solution (Solmedia), rinsed in tap water, dipped twice in acid alcohol solution (1% (v/v) hydrochloric acid, 70% (v/v) ethanol in deionized water), left in running tap water until nuclei were stained blue, stained for 1 h with Picrosirius Red solution (0.1% (w/v) Sirius Red F3B in saturated aqueous picric acid), washed twice with eight dips in a 31% (v/v) solution of acetic acid in deionized water, and then rinsed briefly with tap water. The stained sections were then dehydrated by sequential 30 s immersions in 70% (v/v) ethanol, 90% (v/v) ethanol, 100% (v/v) ethanol, 100% (v/v) ethanol, immersed three times for 5 min in Histoclear, then mounted with coverslips and DPX Mountant. Images were captured using a Leica DMI6000 inverted epifluorescence microscope fitted with a crosspolarizer.

### Immunostaining

The cartilage tissue constructs were washed in PBS, fixed in 4% (v/v) formaldehyde overnight, and then transferred to 70% (v/v) ethanol before paraffin embedding and sectioning (10 µm thick sections). Paraffin was removed using a 6 min immersion in Histoclear before rehydrating the samples by sequential 2 min immersions in 100% (v/v) ethanol, 90% (v/v) ethanol, 70% (v/v) ethanol, and deionized water. Tissue sections were treated for 30 min at 37 °C with a 10 mg mL^–1^ solution of hyaluronidase in PBS, washed for 5 min in PBS, treated for 30 min at 37 °C with a 2 mg mL^–1^ solution of pronase (Boehringer Mannheim) in PBS, washed for 5 min in PBS, treated for 5 min with a 3% (v/v) aqueous solution of hydrogen peroxide, washed for 5 min in PBS, treated for 1 h with a 30 mg mL^–1^ solution of bovine serum albumin (BSA) dissolved in TBS/Tween buffer (6.05 g tris(hydroxymethyl)‐aminomethane, 8.76 g sodium chloride, and 0.5 mL Tween 20 dissolved in 1 mL deionized water, adjusted to pH 7.5–7.6), and then washed three times for 10 min each in TBS/Tween buffer. The sections were then treated overnight at 4 °C with either a goat primary antibody recognizing type II collagen (Cambridge Biosciences) diluted 1:20 or a goat primary antibody recognizing type I collagen (Cambridge Biosciences) diluted 1:200 (antibody diluent: 10 mg mL^–1^ BSA in TBS/Tween buffer). The sections were then washed once for 15 min with high salt buffer (16.2 g L^–1^ sodium chloride in TBS/Tween buffer), washed twice for 15 min each with TBS/Tween buffer, treated for 1 h with a biotinylated rabbit antigoat IgG secondary antibody (from Vectastain Elite ABC‐HRP Kit, Vector Laboratories) diluted 1:200 in antibody diluent, washed once for 15 min with high salt buffer, washed twice for 15 min each with TBS/Tween buffer, then treated for 30 min with Vectastain Elite ABC reagent (comprising 1 drop Reagent A:1 drop Reagent B:2.5 mL TBS/Tween buffer). The sections were then washed three times for 10 min each with TBS/Tween buffer and then treated for 10 min with ImmPACT DAB substrate, peroxidase (2B Scientific). The sections were then washed for 5 min in deionized water, dehydrated by sequential 2 min immersions in 70% (v/v) ethanol, 90% (v/v) ethanol, 100% (v/v) ethanol, immersed twice for 3 min in Histoclear, then mounted with coverslips and DPX Mountant.

### Human Mesenchymal Stem Cell Study

Porcine type A gelatin was modified with methacrylic anhydride to form gelatin methacryloyl with a degree of functionalization of 88 ± 1%. Full details of the reaction, purification, and characterization are described in a previous study.^[^ ^32^ ^]^ Human mesenchymal stem cells (Lonza) were cultured using stem cell culture medium (low glucose DMEM supplemented with 10% (v/v) fetal bovine serum and 10 ng mL^–1^ recombinant human fibroblast growth factor (FGF)‐basic (Peprotech)). To generate a cell suspension for acoustic patterning, hMSCs were briefly washed with PBS, treated for 5 min with Trypsin‐EDTA solution (Gibco) at 37 °C, resuspended in an equivalent volume of stem cell culture medium, centrifuged at 300 × *g* for 5 min, and then resuspended in stem cell culture medium. To provide a nonadherent coating for cell patterning, 2% (w/v) molten UltraPure Agarose 1000 (Thermo Fisher Scientific) was added to the central cavity of an acoustic patterning device and allowed to gel by cooling to room temperature. A suspension of hMSCs in stem cell culture medium was added to an equivalent volume of gelatin methacryloyl dissolved in Irgacure 2959. The final seeding density was 1 × 10^6^ cells mL^–1^ and the final concentrations of gelatin methacryloyl and Irgacure 2959 were 5% (w/v) and 5 mg mL^–1^, respectively. This solution was added to the central cavity of the acoustic patterning device, which was placed on top of a Polar Bear Plus (Cambridge Reactor Design) to maintain the temperature at 37 °C. A single piezotransducer pair was driven at a frequency of 6.6–6.7 MHz and an amplitude of 20 V_pp_ for 10 min. Ultraviolet light was applied for the final 2 min using a filtered lamp (Uvitec) operating at 365 nm and 6 mW cm^–2^. The gelatin methacryloyl hydrogels were then removed from the underlying agarose layer, washed twice with PBS, and then washed once with stem cell culture medium. An unpatterned control was set up with the same procedure but no ultrasound field exposure. The hydrogels were stained for 20 min at 37 °C using a LIVE/DEAD cell viability assay at the recommended dilutions, transferred to phenol‐free DMEM supplemented with 20 × 10^–3^ m HEPES buffer, and then imaged using a Leica SP5 inverted confocal fluorescence microscope.

### Statistical Analysis

i) Preprocessing of data. The data presented in Figure 3a displays the metabolic activity of the two exposed chondrocyte groups (3, 10 min) normalized to the unexposed chondrocytes (0 min). ii) Data presentation. The data presented in Figures 3a,b,d, and S5 (Supporting Information) display individual datapoints with connecting lines denoting data that was obtained from the same biological source. iii) Sample size. Four biological replicates were used for Figures 3a,b,d, while three biological replicates were used for Figure S5 (Supporting Information). iv) Statistical methods. Wilcoxon signed‐rank tests were used to compare groups in Figures 3a,b,d, and S5 (Supporting Information). No comparisons showed any statistically significant difference between groups (*p*‐value > 0.05). v) Software. Origin (OriginLab Corporation) was used for all statistical analyses.

## Conflict of Interest

D.W.H., T.J.K., and C.M. are founders and shareholders of GELOMICS PTY LTD. C.M. is also Managing Director of GELOMICS PTY LTD.

## Supporting information

Supporting Information

Supplemental Video 1

## Data Availability

Research raw data is available upon request from rdm-enquiries@imperial.ac.uk.
