# Supplementary material for: Tissue Engineering Cartilage with Deep Zone Cytoarchitecture by High-Resolution Acoustic Cell Patterning
Source: Adv Healthc Mater. Author manuscript; Available in PMC 2023 Jan 13. (PMC7614068; doi:10.1002/adhm.202200481)
Supplement: Supplementary file 1 [file EMS156638-supplement-Supplementary_Material.zip › adhm202200481-sup-0001-suppmat.pdf]

ADVANCED HEALTHCARE MATERIALS

Supporting Information

for *Adv. Healthcare Mater.*, DOI 10.1002/adhm.202200481

Tissue Engineering Cartilage with Deep Zone Cytoarchitecture by High-Resolution Acoustic Cell Patterning

James P. K. Armstrong, Ekaterina Pchelintseva, Sirli Treumuth, Cristiana Campanella, Christoph Meinert, Travis J. Klein, Dietmar W. Hutmacher, Bruce W. Drinkwater and Molly M. Stevens**

Supporting Information

Tissue engineering cartilage with deep zone cytoarchitecture by high-resolution acoustic cell patterning

James P. K. Armstrong, Ekaterina Pchelintseva, Sirli Treumuth, Cristiana Campanella, Christoph Meinert, Travis J. Klein, Dietmar W. Hutmacher, Bruce W. Drinkwater & Molly M. Stevens**

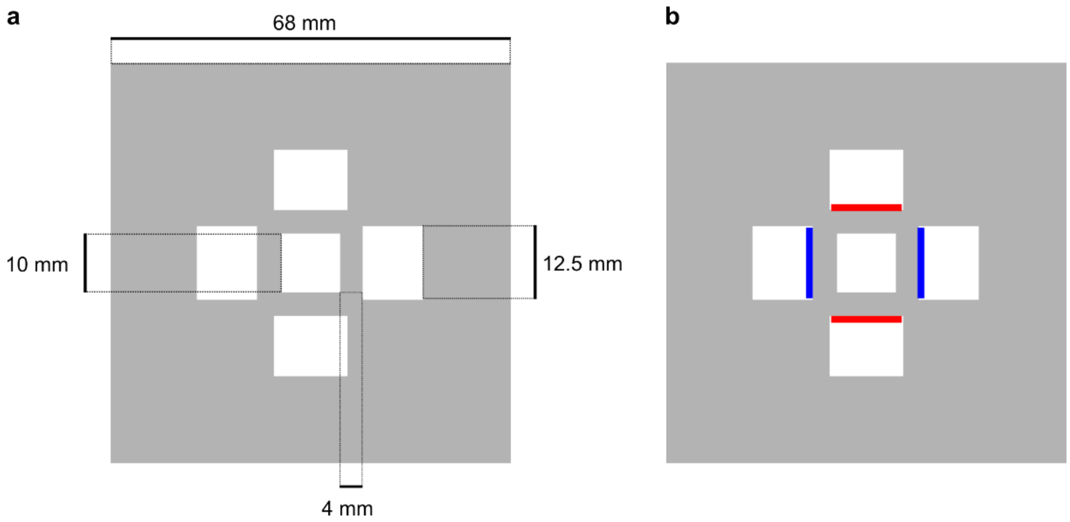

Figure S1. *Design of the acoustic cell patterning device used in this study. (a) 12-mm thick sheets of acrylic (gray) were laser cut to these dimensions to form small units with five cavities. (b) Piezotransducer pairs (shown in red and blue) with wrap-around electrodes were affixed to the inner edge of the four outer cavities. One face of the device is sealed with an acetate sheet and the piezotransducers are connected to a function generator for acoustic cell patterning.*

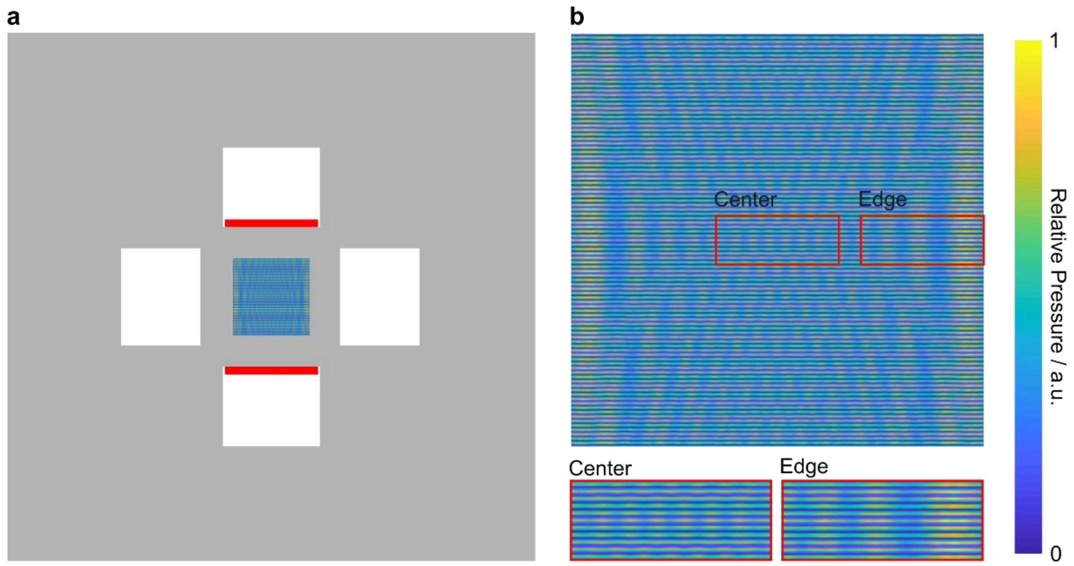

***Figure S2**. Pressure field modeling. The pressure field inside the central cavity of the acoustic patterning device was modeled using Huygens wave theory, accounting for the device geometry and the relative position of the piezotransducers. (a) Schematic showing the piezotransducer pair that is supplying the ultrasound standing wave (red). (b) The pressure field across the whole inner cavity. Inset images show a relatively uniform pressure field at the center of the ultrasound standing wave, with any distortions limited to the edges of the cavity. The color scale shows relative pressure from low pressure (blue) to high pressure (yellow).*

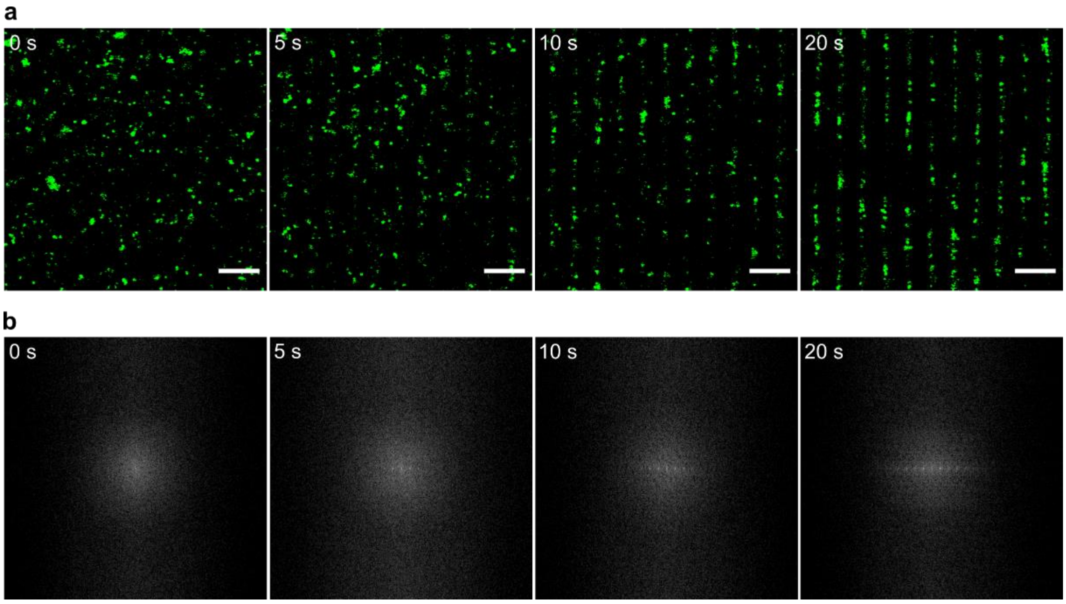

Figure S3. *Fast Fourier transform analysis of acoustic cell patterning. (a) Time-lapse confocal fluorescence microscopy of fixed and fluorescently labeled chondrocytes (green) acoustically patterned in cell culture media using a 6.7 MHz ultrasound standing wave. The chondrocytes rapidly transition from a random distribution to an ordered, linear array. Scale bars = 200 µm. (b) Fast Fourier Transform analysis performed on these micrographs provides a clear visualization of this transition, with a gradual emergence and clarification of periodicity that corresponds to the acoustic cell patterning observed in the fluorescence micrographs.*

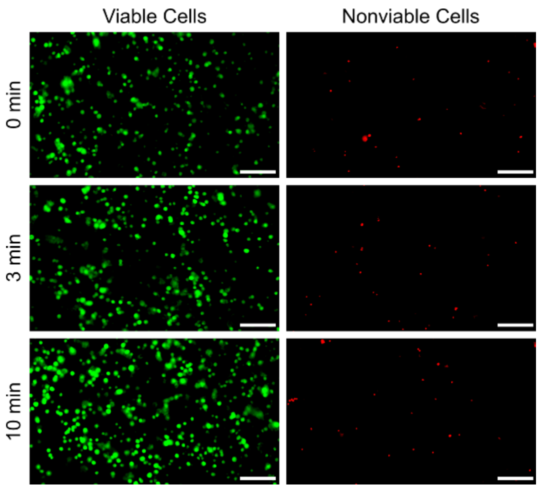

Figure S4. *Membrane viability is preserved following ultrasound exposure. Chondrocytes in culture media were exposed to a 6.7 MHz ultrasound standing wave for either 0, 3, or 10 min, and then immobilized in agarose and cultured for 24 h. Widefield fluorescence microscopy following staining with a LIVE/DEAD assay showed no observable differences in the ratio of viable cells (green) and nonviable cells (red) between the three groups. Scale bars = 100 µm.*

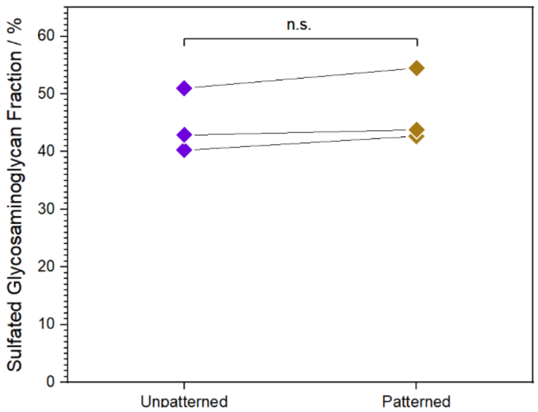

***Figure S5**. Levels of sulfated glycosaminoglycan are similar in patterned and unpatterned cartilage tissues. Agarose hydrogels laden with acoustically patterned chondrocytes were used to engineer cartilage tissue over a 35 d culture period. A dimethylmethylene blue assay showed a high similarity in the level of sulfated glycosaminoglycan between the patterned cartilage and an unpatterned control (Wilcoxon Signed Rank Test, n = 3 with connecting lines indicating that datapoints are obtained from the same biological source, n.s. = nonsignificant).*

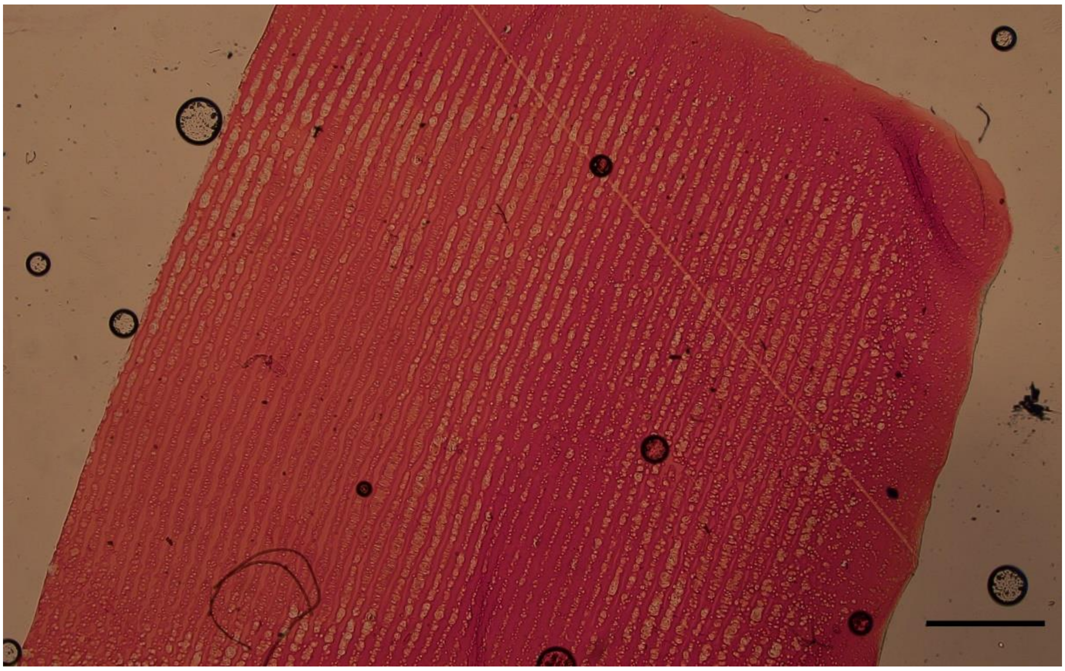

Figure S6. *Low magnification image of engineered cartilage tissue stained with safranin O. Agarose hydrogels were cast with acoustically patterned chondrocytes and used to engineer cartilage tissue, which was sectioned and stained for sulfated glycosaminoglycan using safranin O. Low magnification imaging shows the extent of extracellular matrix deposition and cellular organization in the patterned cartilage tissue. Patterning is present throughout the vast majority of the tissue and is only absent in the outer regions (on the top and right of this section) Note that this tissue was cut prior to sectioning: the cut edge is on the left of this stained section and is not part of the tissue periphery. Scale bar = 1 mm.*

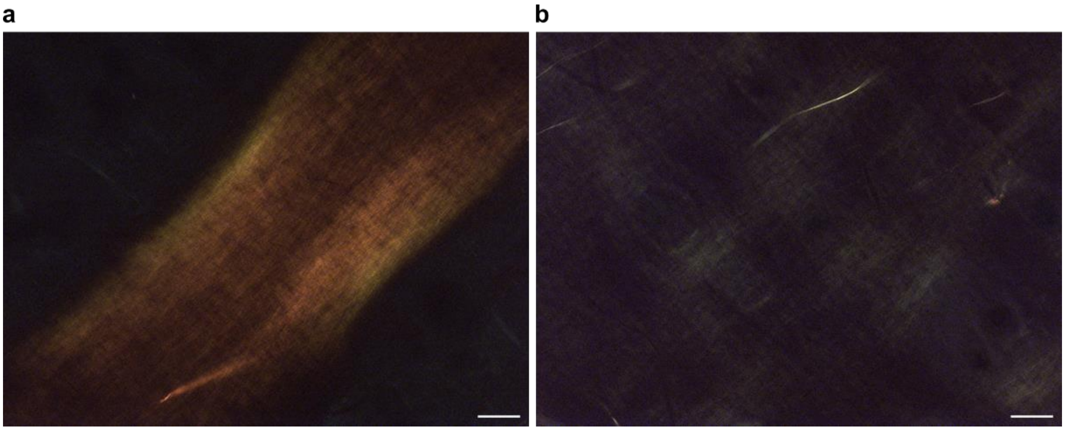

Figure S7. *Polarized microscopy of engineered cartilage tissue stained with picrosirius red. Agarose hydrogels were cast with acoustically patterned chondrocytes and used to engineer cartilage tissue, which was sectioned and stained for collagen using picrosirius red. Polarized microscopy was used to detect the presence of collagen fibers. (a) Patterned cartilage tissue exhibiting oriented collagen fibers co-aligned with the chondrocyte array. (b) Unpatterned cartilage tissue exhibiting a reduced birefringence and no bulk directionality in collagen fiber orientation. Scale bars = 10 µm.*

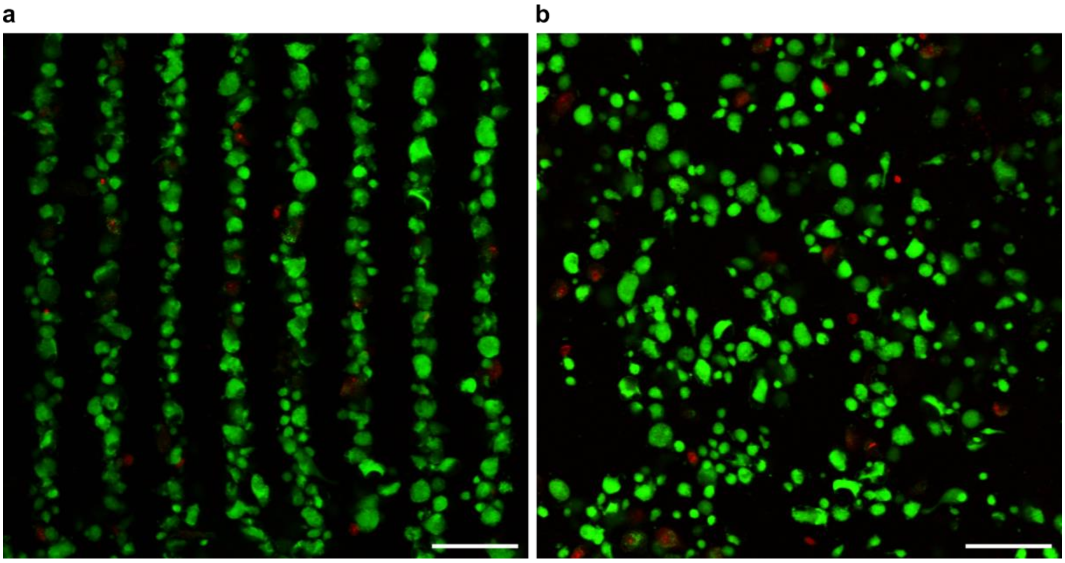

Figure S8. Acoustic patterning of hMSCs in GelMA hydrogels. (a) 5% (w/v) GelMA hydrogels were cast with acoustically patterned hMSCs and stained with a LIVE/DEAD viability assay immediately after gelation. These micrographs reveal a patterned cytoarchitecture similar to the chondrocyte-agarose system. (b) No cell organization was observed in unpatterned controls. Scale bars = 150 µm.
